# Supplementary material for: Development and Validation of a Primary Care Electronic Health Record Phenotype to Study Migration and Health in the UK
Source: Int J Environ Res Public Health. 2021 Dec 17;18(24):13304. doi: 10.3390/ijerph182413304 (PMC8707886; doi:10.3390/ijerph182413304)
Supplement: Supplementary file 1 [file ijerph-18-13304-s001.zip › ijerph-1428637-supplementary.pdf]

Table S1. Code list to create 18 categories of ethnicity, and a 6-category higher level grouping of ethnicity.

6 level grouping	18 level grouping	Read code	Read term	Medcode
White British	White British	9S10.00	White British	12446
White British	White British	9S13.00	White Scottish	26467
White British	White British	9S14.00	Other white British ethnic group	26310
White British	White British	9i00.00	White British - ethnic category 2001 census	98111
White British	White British	9i20.00	English - ethnic category 2001 census	12352
White British	White British	9i21.00	Scottish - ethnic category 2001 census	12436
White British	White British	9i22.00	Welsh - ethnic category 2001 census	12681
White British	White British	9i23.00	Cornish - ethnic category 2001 census	28887
White Non-British	White Irish	9S11.00	White Irish	24837
White Non-British	White Irish	9SA9.00	Irish (NMO)	24270
White Non-British	White Irish	9SI..00	Irish traveller	47601
White Non-British	White Irish	9i1..00	Irish - ethnic category 2001 census	12532
White Non-British	White Irish	9i10.00	White Irish - ethnic category 2001 census	98213
White Non-British	White Irish	9i24.00	Northern Irish - ethnic category 2001 census	42294
White Non-British	White Irish	9i2C.00	Irish Traveller - ethnic category 2001 census	55223
White Non-British	White Other	9S12.00	Other white ethnic group	12444
White Non-British	White Other	9SAA.00	Greek/Greek Cypriot (NMO)	45947
White Non-British	White Other	9SAA.11	Greek (NMO)	45955
White Non-British	White Other	9SAA.12	Greek Cypriot (NMO)	47949
White Non-British	White Other	9SAB.00	Turkish/Turkish Cypriot (NMO)	32066
White Non-British	White Other	9SAB.11	Turkish (NMO)	32126
White Non-British	White Other	9SAB.12	Turkish Cypriot (NMO)	32069
White Non-British	White Other	9SAC.00	Other European (NMO)	12633
White Non-British	White Other	9T1..00	New Zealand ethnic groups	45008
White Non-British	White Other	9T1Y.00	Other New Zealand ethnic group	96789
White Non-British	White Other	9T1Z.00	New Zealand ethnic group NOS	71425
White Non-British	White Other	9i2..00	Other White background - ethnic category 2001 census	12421
White Non-British	White Other	9i26.00	Cypriot (part not stated) - ethnic category 2001 census	32778
White Non-British	White Other	9i27.00	Greek - ethnic category 2001 census	12355
White Non-British	White Other	9i28.00	Greek Cypriot - ethnic category 2001 census	12769
White Non-British	White Other	9i29.00	Turkish - ethnic category 2001 census	12746
White Non-British	White Other	9i2A.00	Turkish Cypriot - ethnic category 2001 census	32413
White Non-British	White Other	9i2B.00	Italian - ethnic category 2001 census	12412
White Non-British	White Other	9i2D.00	Traveller - ethnic category 2001 census	55113
White Non-British	White Other	9i2E.00	Gypsy/Romany - ethnic category 2001 census	42290
White Non-British	White Other	9i2F.00	Polish - ethnic category 2001 census	12467
White Non-British	White Other	9i2G.00	Baltic Estonian/Latvian/Lithuanian - ethn categ 2001 census	12433
White Non-British	White Other	9i2H.00	Commonwealth (Russian) Indep States - ethn categ 2001 census	28973
White Non-British	White Other	9i2J.00	Kosovan - ethnic category 2001 census	26341
White Non-British	White Other	9i2K.00	Albanian - ethnic category 2001 census	25422
White Non-British	White Other	9i2L.00	Bosnian - ethnic category 2001 census	46956
White Non-British	White Other	9i2M.00	Croatian - ethnic category 2001 census	28866
White Non-British	White Other	9i2N.00	Serbian - ethnic category 2001 census	47074
White Non-British	White Other	9i2P.00	Other republics former Yugoslavia - ethnic categ 2001 census	28936
White Non-British	White Other	9i2T.00	Other White or White unspecified ethnic category 2001 census	12591
White Non-British	White NOS	9S1..00	White	22467
White Non-British	White NOS	9SB3.00	Other ethnic, mixed white orig	35459
White Non-British	White NOS	9i25.00	Ulster Scots - ethnic category 2001 census	40102
White Non-British	White NOS	9i2Q.00	Mixed Irish and other White - ethnic category 2001 census	26391
White Non-British	White NOS	9i2R.00	Oth White European/European unsp/Mixed European 2001 census	12402
White Non-British	White NOS	9i2S.00	Other mixed White - ethnic category 2001 census	28900
Mixed	Mixed White and Black	9SB5.00	Black Caribbean and White	32425
Mixed	Mixed White and Black	9SB6.00	Black African and White	32443
Mixed	Mixed White and Black	9i3..00	White and Black Caribbean - ethnic category 2001 census	12742
Mixed	Mixed White and Black	9i4..00	White and Black African - ethnic category 2001 census	12437
Mixed	Mixed White and Asian	9SB2.00	Other ethnic, Asian/White orig	32401
Mixed	Mixed White and Asian	9i5..00	White and Asian - ethnic category 2001 census	12638
Mixed	Mixed Asian and Black	9S45.00	Black E Afric Asia/Indo-Caribb	47965
Mixed	Mixed Asian and Black	9S45.11	Black East African Asian	57753
Mixed	Mixed Asian and Black	9S45.12	Black Indo-Caribbean	57763
Mixed	Mixed Asian and Black	9S46.00	Black Indian sub-continent	48005
Mixed	Mixed Asian and Black	9S47.00	Black - other Asian	35350
Mixed	Mixed Asian and Black	9S52.00	Other Black - Black/Asian orig	32165
Mixed	Mixed Asian and Black	9SA6.00	E Afric Asian/Indo-Carib (NMO)	38097

Mixed	Mixed Asian and Black	9i60.00	Black and Asian - ethnic category 2001 census	12795
Mixed	Mixed Asian and Black	9i61.00	Black and Chinese - ethnic category 2001 census	49940
Mixed	Mixed Asian and Black	9iA7.00	Caribbean Asian - ethnic category 2001 census	32399
Mixed	Mixed Other	9SB..00	Other ethnic, mixed origin	12696
Mixed	Mixed Other	9SB4.00	Other ethnic, other mixed orig	32420
Mixed	Mixed Other	9i6..00	Other Mixed background - ethnic category 2001 census	12873
Mixed	Mixed Other	9i63.00	Chinese and White - ethnic category 2001 census	12706
Mixed	Mixed Other	9i64.00	Asian and Chinese - ethnic category 2001 census	47005
Mixed	Mixed Other	9i65.00	Other Mixed or Mixed unspecified ethnic category 2001 census	32408
Mixed	Mixed NOS	9S51.00	Other Black - Black/White orig	25623
Mixed	Mixed NOS	9SB1.00	Other ethnic, Black/White orig	47401
Mixed	Mixed NOS	9i62.00	Black and White - ethnic category 2001 census	40110
Asian/Asian British	Indian	9S6..00	Indian	12482
Asian/Asian British	Indian	9SA7.00	Indian sub-continent (NMO)	39696
Asian/Asian British	Indian	9i7..00	Indian or British Indian - ethnic category 2001 census	12414
Asian/Asian British	Indian	9iA1.00	Punjabi - ethnic category 2001 census	26392
Asian/Asian British	Pakistani	9S7..00	Pakistani	24690
Asian/Asian British	Pakistani	9i8..00	Pakistani or British Pakistani - ethnic category 2001 census	12460
Asian/Asian British	Pakistani	9iA2.00	Kashmiri - ethnic category 2001 census	64133
Asian/Asian British	Bangladeshi	9S8..00	Bangladeshi	24740
Asian/Asian British	Bangladeshi	9i9..00	Bangladeshi or British Bangladeshi - ethn categ 2001 census	28888
Asian/Asian British	Other Asian	9SA6.11	East African Asian (NMO)	46818
Asian/Asian British	Other Asian	9SA8.00	Other Asian (NMO)	26379
Asian/Asian British	Other Asian	9SH..00	Other Asian ethnic group	12668
Asian/Asian British	Other Asian	9iA..00	Other Asian background - ethnic category 2001 census	12513
Asian/Asian British	Other Asian	9iA3.00	East African Asian - ethnic category 2001 census	47077
Asian/Asian British	Other Asian	9iA8.00	British Asian - ethnic category 2001 census	12653
Asian/Asian British	Other Asian	9iA9.00	Mixed Asian - ethnic category 2001 census	46056
Asian/Asian British	Other Asian	9iAA.00	Other Asian or Asian unspecified ethnic category 2001 census	28935
Asian/Asian British	Other Asian	9SA6.12	Indo-Caribbean (NMO)	99316
Black/Black British	Black Caribbean	9S2..00	Black Caribbean	12632
Black/Black British	Black Caribbean	9S42.00	Black Caribbean/W.I./Guyana	57435
Black/Black British	Black Caribbean	9S42.11	Black Caribbean	47950
Black/Black British	Black Caribbean	9S42.12	Black West Indian	47997
Black/Black British	Black Caribbean	9S42.13	Black Guyana	32100
Black/Black British	Black Caribbean	9SA3.00	Caribbean I./W.I./Guyana (NMO)	54593
Black/Black British	Black Caribbean	9SA3.11	Caribbean Island (NMO)	57094
Black/Black British	Black Caribbean	9SA3.12	West Indian (NMO)	57075
Black/Black British	Black Caribbean	9SA3.13	Guyana (NMO)	93144
Black/Black British	Black Caribbean	9iB..00	Caribbean - ethnic category 2001 census	12432
Black/Black British	Black African	9S3..00	Black African	12778
Black/Black British	Black African	9S44.00	Black - other African country	35412
Black/Black British	Black African	9SA5.00	Other African countries (NMO)	47969
Black/Black British	Black African	9iC..00	African - ethnic category 2001 census	12350
Black/Black British	Black African	9iD0.00	Somali - ethnic category 2001 census	12443
Black/Black British	Black African	9iD1.00	Nigerian - ethnic category 2001 census	32886
Black/Black British	Black Other	9S4..00	Black, other, non-mixed origin	24339
Black/Black British	Black Other	9S41.00	Black British	12452
Black/Black British	Black Other	9S43.00	Black N African/Arab/Iranian	41329
Black/Black British	Black Other	9S43.11	Black North African	46812
Black/Black British	Black Other	9S43.12	Black Arab	57752
Black/Black British	Black Other	9S43.13	Black Iranian	50286
Black/Black British	Black Other	9S48.00	Black Black - other	26312
Black/Black British	Black Other	9S5..00	Black - other, mixed	25676
Black/Black British	Black Other	9SG..00	Other black ethnic group	32136
Black/Black British	Black Other	9iD..00	Other Black background - ethnic category 2001 census	32389
Black/Black British	Black Other	9iD2.00	Black British - ethnic category 2001 census	40097
Black/Black British	Black Other	9iD3.00	Mixed Black - ethnic category 2001 census	40096
Black/Black British	Black Other	9iD4.00	Other Black or Black unspecified ethnic category 2001 census	46047
Other	Chinese	9S9..00	Chinese	24272
Other	Chinese	9iE..00	Chinese - ethnic category 2001 census	12468
Other	Other ethnic group	9SA..00	Other ethnic non-mixed (NMO)	30280
Other	Other ethnic group	9SA1.00	Brit. ethnic minor. spec.(NMO)	32110
Other	Other ethnic group	9SA2.00	Brit. ethnic minor. unsp (NMO)	57764
Other	Other ethnic group	9SA4.00	N African Arab/Iranian (NMO)	24962
Other	Other ethnic group	9SA4.11	North African Arab (NMO)	47285
Other	Other ethnic group	9SA4.12	Iranian (NMO)	25082
Other	Other ethnic group	9SAD.00	Other ethnic NEC (NMO)	41214
Other	Other ethnic group	9SC..00	Vietnamese	25411
Other	Other ethnic group	9SJ..00	Other ethnic group	12757

Other	Other ethnic group	9T1A.00	Other Pacific ethnic group	46752
Other	Other ethnic group	9iA4.00	Sri Lankan - ethnic category 2001 census	12608
Other	Other ethnic group	9iA5.00	Tamil - ethnic category 2001 census	12760
Other	Other ethnic group	9iA6.00	Sinhalese - ethnic category 2001 census	12887
Other	Other ethnic group	9iF..00	Other - ethnic category 2001 census	12434
Other	Other ethnic group	9iF0.00	Vietnamese - ethnic category 2001 census	12719
Other	Other ethnic group	9iF1.00	Japanese - ethnic category 2001 census	12473
Other	Other ethnic group	9iF2.00	Filipino - ethnic category 2001 census	12420
Other	Other ethnic group	9iF3.00	Malaysian - ethnic category 2001 census	12730
Other	Other ethnic group	9iF4.00	Buddhist - ethnic category 2001 census	63872
Other	Other ethnic group	9iF5.00	Hindu - ethnic category 2001 census	56127
Other	Other ethnic group	9iF6.00	Jewish - ethnic category 2001 census	46063
Other	Other ethnic group	9iF7.00	Muslim - ethnic category 2001 census	47091
Other	Other ethnic group	9iF8.00	Sikh - ethnic category 2001 census	49658
Other	Other ethnic group	9iF9.00	Arab - ethnic category 2001 census	46059
Other	Other ethnic group	9iFA.00	North African - ethnic category 2001 census	47028
Other	Other ethnic group	9iFB.00	Mid East (excl Israeli, Iranian & Arab) - eth cat 2001 cens	28909
Other	Other ethnic group	9iFC.00	Israeli - ethnic category 2001 census	46964
Other	Other ethnic group	9iFD.00	Iranian - ethnic category 2001 census	25937
Other	Other ethnic group	9iFE.00	Kurdish - ethnic category 2001 census	45964
Other	Other ethnic group	9iFF.00	Moroccan - ethnic category 2001 census	25451
Other	Other ethnic group	9iFG.00	Latin American - ethnic category 2001 census	26246
Other	Other ethnic group	9iFH.00	South and Central American - ethnic category 2001 census	12756
Other	Other ethnic group	9iFJ.00	Mauritian/Seychellois/Maldivian/St Helena eth cat 2001census	32382
Other	Other ethnic group	9iFK.00	Any other group - ethnic category 2001 census	26455
N/A	Ethnic group not specified	916E.00	Patient ethnicity unknown	93749
N/A	Ethnic group not specified	9S...00	Ethnic groups (1991 census)	10196
N/A	Ethnic group not specified	9SD..00	Ethnic group not given - patient refused	12429
N/A	Ethnic group not specified	9SE..00	Ethnic group not recorded	24340
N/A	Ethnic group not specified	9SZ..00	Ethnic groups (census) NOS	45199
N/A	Ethnic group not specified	9T...00	Ethnicity and other related nationality data	23955
N/A	Ethnic group not specified	9i...00	Ethnic category - 2001 census	12435
N/A	Ethnic group not specified	9i0..00	British or mixed British - ethnic category 2001 census	12351
N/A	Ethnic group not specified	9iG..00	Ethnic category not stated - 2001 census	12459

Table S2. Migration phenotype, including Read (V2) codes, Read terms, Medcodes, type of code and level of certainty of migration status.

Level of certainty of migration status	Type of code	Read code	Read term	Medcode
Definite	Born outside of the UK	13e..00	Country of birth (Asian)	11552
Definite	Born outside of the UK	13gf.00	Born in South Africa	12458
Definite	Born outside of the UK	13eG.00	Born in Iraq	12713
Definite	Born outside of the UK	13eH.00	Born in Israel	25007
Definite	Born outside of the UK	13go.00	Born in Zimbabwe	25008
Definite	Born outside of the UK	13eY.00	Born in Philippines	25092
Definite	Born outside of the UK	13gi.00	Born in Tanzania	25133
Definite	Born outside of the UK	13dl.00	Born in Yugoslavia	25256
Definite	Born outside of the UK	13gC.00	Born in Congo	25664
Definite	Born outside of the UK	13dM.00	Born in Kosovo	25730
Definite	Born outside of the UK	13eW.00	Born in Pakistan	25995
Definite	Born outside of the UK	13eo.00	Born in Vietnam	26334
Definite	Born outside of the UK	13eF.00	Born in Iran	26426
Definite	Born outside of the UK	13dA.00	Born in Czech Republic	26463
Definite	Born outside of the UK	13dP.00	Born in Lithuania	28301
Definite	Born outside of the UK	13eg.00	Born in Syria	28529
Definite	Born outside of the UK	13gY.00	Born in Niger	30224
Definite	Born outside of the UK	13f5.00	Born in Canada	30606
Definite	Born outside of the UK	13f..00	Country of birth (American)	32053
Definite	Born outside of the UK	13d0.00	Born in Albania	32055
Definite	Born outside of the UK	13e8.00	Born in China	32058
Definite	Born outside of the UK	13e0.00	Born in Afghanistan	32060
Definite	Born outside of the UK	13h0.00	Born in Australia	32061
Definite	Born outside of the UK	13jC.00	Born in Trinidad and Tobago	32062
Definite	Born outside of the UK	13gJ.00	Born in Ghana	32067
Definite	Born outside of the UK	13gl.00	Born in Uganda	32068
Definite	Born outside of the UK	13eI.00	Born in Japan	32070
Definite	Born outside of the UK	13fL.00	Born in USA	32072
Definite	Born outside of the UK	13h1.00	Born in New Zealand	32074
Definite	Born outside of the UK	13f3.00	Born in Brazil	32075
Definite	Born outside of the UK	13j6.00	Born in Jamaica	32076
Definite	Born outside of the UK	13g0.00	Born in Algeria	32079
Definite	Born outside of the UK	13dH.00	Born in Greece	32080
Definite	Born outside of the UK	13dF.00	Born in France	32081
Definite	Born outside of the UK	13eD.00	Born in India	32082
Definite	Born outside of the UK	13dG.00	Born in Germany	32085
Definite	Born outside of the UK	13j2.00	Born in Barbados	32089
Definite	Born outside of the UK	13fN.00	Born in Venezuela	32090
Definite	Born outside of the UK	13dW.00	Born in Poland	32094
Definite	Born outside of the UK	13de.00	Born in Spain	32097
Definite	Born outside of the UK	13df.00	Born in Sweden	32098
Definite	Born outside of the UK	13gM.00	Born in Ivory Coast	32099
Definite	Born outside of the UK	13g..00	Country of birth (African)	32102
Definite	Born outside of the UK	13di.00	Born in Ukraine	32103
Definite	Born outside of the UK	13gV.00	Born in Morocco	32108
Definite	Born outside of the UK	13gZ.00	Born in Nigeria	32111
Definite	Born outside of the UK	13gN.00	Born in Kenya	32112
Definite	Born outside of the UK	13dK.00	Born in Ireland	32113
Definite	Born outside of the UK	13ek.00	Born in Turkey	32114
Definite	Born outside of the UK	13gS.00	Born in Malawi	32115
Definite	Born outside of the UK	13dL.00	Born in Italy	32116
Definite	Born outside of the UK	13dc.00	Born in Slovakia	32119
Definite	Born outside of the UK	13fB.00	Born in Grenada	32120
Definite	Born outside of the UK	13e3.00	Born in Bangladesh	32125
Definite	Born outside of the UK	13eb.00	Born in Russia	32127
Definite	Born outside of the UK	13eM.00	Born in Kyrgyzstan	32128
Definite	Born outside of the UK	13j9.00	Born in St. Lucia	32131
Definite	Born outside of the UK	13ej.00	Born in Thailand	32135
Definite	Born outside of the UK	13dD.00	Born in Estonia	32139
Definite	Born outside of the UK	13gU.00	Born in Mauritius	32140
Definite	Born outside of the UK	13ec.00	Born in Saudi Arabia	32144

Definite	Born outside of the UK	13dh.00	Born in The Netherlands	32150
Definite	Born outside of the UK	13ed.00	Born in Singapore	32157
Definite	Born outside of the UK	13gG.00	Born in Ethiopia	32158
Definite	Born outside of the UK	13dX.00	Born in Portugal	32160
Definite	Born outside of the UK	13gW.00	Born in Mozambique	32162
Definite	Born outside of the UK	13fJ.00	Born in Peru	32166
Definite	Born outside of the UK	13g5.00	Born in Burundi	32167
Definite	Born outside of the UK	13gn.00	Born in Zambia	32168
Definite	Born outside of the UK	13d7.00	Born in Bulgaria	32169
Definite	Born outside of the UK	13eP.00	Born in Malaysia	32171
Definite	Born outside of the UK	13dE.00	Born in Finland	32173
Definite	Born outside of the UK	13dB.00	Born in Denmark	32186
Definite	Born outside of the UK	13ge.00	Born in Somalia	32189
Definite	Born outside of the UK	13d9.00	Born in Cyprus	32190
Definite	Born outside of the UK	13gd.00	Born in Sierra Leone	32197
Definite	Born outside of the UK	13fF.00	Born in Mexico	32201
Definite	Born outside of the UK	13e7.00	Born in Chechnya	32202
Definite	Born outside of the UK	13gI.00	Born in Gambia	32207
Definite	Born outside of the UK	13eT.00	Born in Nepal	32217
Definite	Born outside of the UK	13eC.00	Born in Hong Kong	32220
Definite	Born outside of the UK	13gX.00	Born in Namibia	32233
Definite	Born outside of the UK	13gP.00	Born in Liberia	32237
Definite	Born outside of the UK	13dN.00	Born in Latvia	32242
Definite	Born outside of the UK	13ef.00	Born in Sri Lanka	32245
Definite	Born outside of the UK	13e6.00	Born in Burma	32254
Definite	Born outside of the UK	13g7.00	Born in Cameroon	32255
Definite	Born outside of the UK	13g1.00	Born in Angola	32260
Definite	Born outside of the UK	13e2.00	Born in Bahrain	32273
Definite	Born outside of the UK	13k4.00	Born in Seychelles	32293
Definite	Born outside of the UK	13j0.00	Born in Antigua and Barbuda	32301
Definite	Born outside of the UK	13f7.00	Born in Columbia	32303
Definite	Born outside of the UK	13jB.00	Born in Togo	32304
Definite	Born outside of the UK	13gc.00	Born in Senegal	32309
Definite	Born outside of the UK	13f9.00	Born in Ecuador	32311
Definite	Born outside of the UK	13d2.00	Born in Austria	32313
Definite	Born outside of the UK	13f0.00	Born in Argentina	32325
Definite	Born outside of the UK	13ga.00	Born in Rwanda	32331
Definite	Born outside of the UK	13gE.00	Born in Egypt	32333
Definite	Born outside of the UK	13f4.00	Born in British Guyana	32342
Definite	Born outside of the UK	13dZ.00	Born in Romania	32345
Definite	Born outside of the UK	13gL.00	Born in Guinea Republic	32347
Definite	Born outside of the UK	13d4.00	Born in Belgium	32352
Definite	Born outside of the UK	13e1.00	Born in Armenia	32361
Definite	Born outside of the UK	13j4.00	Born in Dominican Republic	32369
Definite	Born outside of the UK	13eh.00	Born in Taiwan	32390
Definite	Born outside of the UK	13d6.00	Born in Bosnia - Herzegovnia	32397
Definite	Born outside of the UK	13k..00	Country of birth (Pacific)	32417
Definite	Born outside of the UK	13dI.00	Born in Hungary	32688
Definite	Born outside of the UK	13gk.00	Born in Tunisia	32741
Definite	Born outside of the UK	13dg.00	Born in Switzerland	32807
Definite	Born outside of the UK	13gg.00	Born in Sudan	36794
Definite	Born outside of the UK	13gR.00	Born in Madagascar	37197
Definite	Born outside of the UK	13fD.00	Born in Guyana	38075
Definite	Born outside of the UK	13gA.00	Born in Chad	38117
Definite	Born outside of the UK	13dS.00	Born in Moldavia	39974
Definite	Born outside of the UK	13gm.00	Born in Zaire	41209
Definite	Born outside of the UK	13d8.00	Born in Croatia	41210
Definite	Born outside of the UK	13eO.00	Born in Lebanon	41211
Definite	Born outside of the UK	13dV.00	Born in Norway	41213
Definite	Born outside of the UK	13eL.00	Born in Kuwait	41217
Definite	Born outside of the UK	13ee.00	Born in South Korea	41228
Definite	Born outside of the UK	13f2.00	Born in Bolivia	41230
Definite	Born outside of the UK	13d5.00	Born in Belorussia	41233
Definite	Born outside of the UK	13fE.00	Born in Honduras	41280
Definite	Born outside of the UK	13eE.00	Born in Indonesia	41289
Definite	Born outside of the UK	13j3.00	Born in Cuba	41290
Definite	Born outside of the UK	13f6.00	Born in Chile	41291
Definite	Born outside of the UK	13eK.00	Born in Kazakhstan	41292
Definite	Born outside of the UK	13eX.00	Born in Palestine	41297
Definite	Born outside of the UK	13el.00	Born in Turkmenistan	41302

Definite	Born outside of the UK	13dd.00	Born in Slovenia	41304
Definite	Born outside of the UK	13eJ.00	Born in Jordan	41311
Definite	Born outside of the UK	13dJ.00	Born in Iceland	41312
Definite	Born outside of the UK	13g3.00	Born in Botswana	41316
Definite	Born outside of the UK	13g2.00	Born in Benin	41318
Definite	Born outside of the UK	13h..00	Country of birth (Australasian)	41327
Definite	Born outside of the UK	13d3.00	Born in Azerbaijan	41337
Definite	Born outside of the UK	13gQ.00	Born in Libya	41341
Definite	Born outside of the UK	13ep.00	Born in Yemen	41344
Definite	Born outside of the UK	13gh.00	Born in Swaziland	41350
Definite	Born outside of the UK	13eS.00	Born in Mongolia	41351
Definite	Born outside of the UK	13e9.00	Born in Democratic People's Republic of Korea	41354
Definite	Born outside of the UK	13ea.00	Born in Republic of Korea	41356
Definite	Born outside of the UK	13dY.00	Born in Republic of Ireland	41357
Definite	Born outside of the UK	13dR.00	Born in Malta	41364
Definite	Born outside of the UK	13gK.00	Born in Guinea Bissau	41365
Definite	Born outside of the UK	13fM.00	Born in Uruguay	41367
Definite	Born outside of the UK	13em.00	Born in United Arab Emirates	41372
Definite	Born outside of the UK	13fI.00	Born in Paraguay	41399
Definite	Born outside of the UK	13en.00	Born in Uzbekistan	41402
Definite	Born outside of the UK	13k0.00	Born in Fiji	42635
Definite	Born outside of the UK	13eZ.00	Born in Qatar	42639
Definite	Born outside of the UK	13k6.00	Born in Tonga	47559
Definite	Born outside of the UK	13k5.00	Born in Solomon Islands	48297
Definite	Born outside of the UK	13eB.00	Born in Georgia	49402
Definite	Born outside of the UK	13j..00	Country of birth (Atlantic)	49907
Definite	Born outside of the UK	13eV.00	Born in Oman	51778
Definite	Born outside of the UK	13eA.00	Born in East Timor	57186
Definite	Born outside of the UK	13k7.00	Born in Tuvalu	57189
Definite	Born outside of the UK	13j5.00	Born in Haiti	58192
Definite	Born outside of the UK	13g4.00	Born in Burkina Faso	58527
Definite	Born outside of the UK	13j1.00	Born in Bahamas	58533
Definite	Born outside of the UK	13gH.00	Born in Gabon	59657
Definite	Born outside of the UK	13e5.00	Born in Brunei	62298
Definite	Born outside of the UK	13fH.00	Born in Panama	63923
Definite	Born outside of the UK	13gj.00	Born in The Gambia	63927
Definite	Born outside of the UK	13fA.00	Born in El Salvador	63943
Definite	Born outside of the UK	13k3.00	Born in Papua New Guinea	64120
Definite	Born outside of the UK	13gp.00	Born in Eritrea	64949
Definite	Born outside of the UK	13f8.00	Born in Costa Rica	64984
Definite	Born outside of the UK	13eR.00	Born in Mali	65310
Definite	Born outside of the UK	13jA.00	Born in St. Vincent	66551
Definite	Born outside of the UK	13j8.00	Born in St. Kitts and Nevis	66553
Definite	Born outside of the UK	13eU.00	Born in North Korea	66560
Definite	Born outside of the UK	13gD.00	Born in Djibouti	68866
Definite	Born outside of the UK	13dQ.00	Born in Luxembourg	69131
Definite	Born outside of the UK	13j7.00	Born in Puerto Rico	69135
Definite	Born outside of the UK	13gT.00	Born in Mauritania	69143
Definite	Born outside of the UK	13fK.00	Born in Suriname	69426
Definite	Born outside of the UK	13eN.00	Born in Laos	69431
Definite	Born outside of the UK	13g8.00	Born in Cape Verde Islands	69560
Definite	Born outside of the UK	13fC.00	Born in Guatemala	69806
Definite	Born outside of the UK	13dT.00	Born in Monaco	71190
Definite	Born outside of the UK	13fG.00	Born in Nicaragua	74892
Definite	Born outside of the UK	13eQ.00	Born in Maldives	91328
Definite	Born outside of the UK	13gO.00	Born in Lesotho	93697
Definite	Born outside of the UK	13gF.00	Born in Equatorial Guinea	93923
Definite	Born outside of the UK	13d1.00	Born in Andorra	93935
Definite	Born outside of the UK	13f1.00	Born in Belize	94050
Definite	Born outside of the UK	13dO.00	Born in Liechtenstein	95708
Definite	Born outside of the UK	13t..00	Born in British overseas territory	96295
Definite	Born outside of the UK	13g6.00	Born in Cambodia	96636
Definite	Born outside of the UK	13dm.00	Born in former Yugoslav Republic of Macedonia	96824
Definite	Born outside of the UK	13e4.00	Born in Bhutan	97390
Definite	Born outside of the UK	13t1.00	Born in Bermuda	98038
Definite	Born outside of the UK	13da.00	Born in San Marino	98530
Definite	Born outside of the UK	13dj.00	Born in Vatican City	99119
Definite	Born outside of the UK	13g9.00	Born in Central African Republic	99258
Definite	Born outside of the UK	13jD.00	Born in Dominica	99431
Definite	Born outside of the UK	13ei.00	Born in Tajikistan	100007

Definite	Born outside of the UK	13dn.00	Born in Serbia	100517
Definite	Born outside of the UK	13gB.00	Born in Comoros Islands	101158
Definite	Born outside of the UK	13gb.00	Born in Sao Tome and Principe	101591
Definite	Born outside of the UK	13Zq.00	Country of birth unknown	101846
Definite	Born outside of the UK	13v0.00	Born in Martinique	103364
Definite	Born outside of the UK	13k9.00	Born in Western Samoa	103965
Definite	Born outside of the UK	13t2.00	Born in Anguilla	104983
Definite	Born outside of the UK	13t0.00	Born in Montserrat	105923
Definite	Born outside of the UK	13jE.00	Born in Aruba	108271
Definite	Born outside of the UK	13v7.00	Born in Guadeloupe	108936
Definite	Born outside of the UK	13eq.00	Born in Christmas Island	109226
Definite	Born outside of the UK	13dq.00	Born in Republic of Moldova	109260
Definite	Born outside of the UK	13t5.00	Born in Saint Helena, Ascension and Tristan da Cunha	109276
Definite	Born outside of the UK	13do.00	Born in Montenegro	109457
Definite	Born outside of the UK	13jG.00	Born in Saint Vincent and the Grenadines	109458
Definite	Born outside of the UK	13t3.00	Born in British Virgin Islands	109727
Definite	Born outside of the UK	13kB.00	Born in American Samoa	109791
Definite	Born outside of the UK	13dp.00	Born in Belarus	109898
Definite	Born outside of the UK	13gq.00	Born in Democratic Republic of Congo	109992
Definite	Born outside of the UK	13v4.00	Born in French Guiana	111051
Definite	Born outside of the UK	13k8.00	Born in Vanuatu	111392
Definite	Born outside of the UK	13jH.00	Born in Sint Maarten	111700
Definite	Born outside of the UK	13dx.00	Born in Aland Islands	111774
Definite	Born outside of the UK	13t9.00	Born in Cayman Islands	112269
Definite	Visa status indicating migration	13ZC.00	Immigrant	4114
Definite	Visa status indicating migration	ZV70314	[V]Immigration medical	8929
Definite	Visa status indicating migration	133L.00	Immigrant	9292
Definite	Visa status indicating migration	13ZN.00	Asylum seeker	9627
Definite	Visa status indicating migration	13D3.00	Social migrant	23398
Definite	Visa status indicating migration	13ZB.00	Refugee	24403
Definite	Visa status indicating migration	ZV70516	[V]Refugee health examination	25632
Definite	Visa status indicating migration	13D4.00	Illegal migrant	44407
Definite	Visa status indicating migration	133Q.00	Family reunion immigrant	47073
Definite	Visa status indicating migration	912H.00	Overseas visitor	47513
Definite	Visa status indicating migration	6951	Immigration examination	48029
Definite	Visa status indicating migration	69D8.00	Exam. of refugee	65503
Definite	Visa status indicating migration	13Zd.00	Failed asylum seeker	94906
Definite	Visa status indicating migration	13Zw.00	Has United Kingdom student visa	104123
Definite	Visa status indicating migration	918z.00	Has United Kingdom general visitor visa	107572
Probable	First/main language not English	13lZ.00	Main spoken language Turkish	22294
Probable	First/main language not English	041E.11	Language interpreter	22515
Probable	First/main language not English	13Z6000	English as a second language	23523
Probable	First/main language not English	13Z6500	Language Punjabi	24295
Probable	First/main language not English	13Z6300	Language Hindi	24296
Probable	First/main language not English	13Z6600	Language Urdu	24691
Probable	First/main language not English	13Z6200	Language Gujurati	24712
Probable	First/main language not English	13Z6100	Language Bengali	24741
Probable	First/main language not English	13lC.00	Main spoken language Polish	24881
Probable	First/main language not English	13b0.00	Vietnamese language	25410
Probable	First/main language not English	13lS.00	Main spoken language Albanian	25423
Probable	First/main language not English	13l2.00	Main spoken language Cantonese	25472
Probable	First/main language not English	13lx.00	Main spoken language Thai	25609
Probable	First/main language not English	13lp.00	Main spoken language Malayalam	25616
Probable	First/main language not English	13l5.00	Main spoken language French	25665
Probable	First/main language not English	13l1.00	Main spoken language Bengali	25802
Probable	First/main language not English	13lE.00	Main spoken language Punjabi	25829
Probable	First/main language not English	13lP.00	Main spoken language Shona	26078
Probable	First/main language not English	13Z6400	Language Pashtu	26196
Probable	First/main language not English	13lH.00	Main spoken language Spanish	26247
Probable	First/main language not English	13lb.00	Main spoken language Vietnamese	26335
Probable	First/main language not English	13l0.00	Main spoken language Arabic	26337
Probable	First/main language not English	13lL.00	Main spoken language Urdu	26361
Probable	First/main language not English	13l3.00	Main spoken language Czech	26464
Probable	First/main language not English	13lF.00	Main spoken language Russian	32427
Probable	First/main language not English	13lG.00	Main spoken language Somali	32456
Probable	First/main language not English	13lB.00	Main spoken language Mandarin	32776
Probable	First/main language not English	13lW.00	Main spoken language Japanese	36852
Probable	First/main language not English	13lt.00	Main spoken language Serbian	36862
Probable	First/main language not English	13lQ.00	Main spoken language Italian	36980
Probable	First/main language not English	13lN.00	Main spoken language Kurdish	46014
Probable	First/main language not English	13l8.00	Main spoken language Hindi	46029
Probable	First/main language not English	13lY.00	Main spoken language Lithuanian	46325

Probable	First/main language not English	13lK.00	Main spoken language Tamil	46861
Probable	First/main language not English	13lV.00	Main spoken language Greek	46974
Probable	First/main language not English	13li.00	Main spoken language French Creole	47007
Probable	First/main language not English	13lO.00	Main spoken language Farsi	47029
Probable	First/main language not English	13lJ.00	Main spoken language Sylheti	47627
Probable	First/main language not English	13ld.00	Main spoken language Amharic	47628
Probable	First/main language not English	13lR.00	Main spoken language German	47630
Probable	First/main language not English	13lw.00	Main spoken language Tagalog	47631
Probable	First/main language not English	13lI.00	Main spoken language Swahili	47641
Probable	First/main language not English	13lu.00	Main spoken language Sinhala	47643
Probable	First/main language not English	13lm.00	Main spoken language Igbo	47646
Probable	First/main language not English	13l6.00	Main spoken language Gujerati	48002
Probable	First/main language not English	13b4.00	Mirpuri language	52200
Probable	First/main language not English	13lM.00	Main spoken language Yoruba	54409
Probable	First/main language not English	13lT.00	Main spoken language Croatian	54410
Probable	First/main language not English	13lc.00	Main spoken language Akan	54413
Probable	First/main language not English	13lf.00	Main spoken language Dutch	54414
Probable	First/main language not English	13lX.00	Main spoken language Korean	54415
Probable	First/main language not English	13l9.00	Main spoken language Iba	54416
Probable	First/main language not English	13lv.00	Main spoken language Swedish	54417
Probable	First/main language not English	13lh.00	Main spoken language Flemish	56879
Probable	First/main language not English	13b3.00	Creole language	57462
Probable	First/main language not English	13ll.00	Main spoken language Hebrew	57755
Probable	First/main language not English	13lq.00	Main spoken language Norwegian	57758
Probable	First/main language not English	13l7.00	Main spoken language Hausa	58193
Probable	First/main language not English	13lr.00	Main spoken language Pashto	58643
Probable	First/main language not English	13sA.00	English as a second language	63932
Probable	First/main language not English	13ls.00	Main spoken language Patois	64948
Probable	First/main language not English	13la.00	Main spoken language Ukrainian	66685
Probable	First/main language not English	13lg.00	Main spoken language Ethiopian	69139
Probable	First/main language not English	13lo.00	Main spoken language Luganda	69153
Probable	First/main language not English	13lA.00	Main spoken language Kutchi	72379
Probable	First/main language not English	13lk.00	Main spoken language Hakka	91422
Probable	First/main language not English	ZV60012	[V]Social migrant	93643
Probable	First/main language not English	13u0.00	Main spoken language Bulgarian	95897
Probable	First/main language not English	13ur.00	Main spoken language Latvian	95940
Probable	First/main language not English	13lT.11	Main spoken language Serbo-Croatian	95968
Probable	First/main language not English	13lt.11	Main spoken language Serbo-Croatian	95969
Probable	First/main language not English	13lO.11	Main spoken language Persian	95970
Probable	First/main language not English	13lu.11	Main spoken language Sinhalese	95974
Probable	First/main language not English	13w1.00	Main spoken language Nepali	95978
Probable	First/main language not English	13ua.00	Main spoken language Hungarian	96041
Probable	First/main language not English	13lE.11	Main spoken language Panjabi	96147
Probable	First/main language not English	13u5.00	Main spoken language Afrikaans	96148
Probable	First/main language not English	13u1.00	Main spoken language Romanian	96152
Probable	First/main language not English	13wL.00	Main spoken language Telugu	96163
Probable	First/main language not English	13wR.00	Main spoken language Twi	96223
Probable	First/main language not English	13wG.00	Main spoken language Slovenian	96230
Probable	First/main language not English	13wD.00	Main spoken language Sindhi	96240
Probable	First/main language not English	13ux.00	Main spoken language Marathi	96267
Probable	First/main language not English	13uj.00	Main spoken language Kannada	96268
Probable	First/main language not English	13uN.00	Main spoken language Danish	96289
Probable	First/main language not English	13u6.00	Main spoken language Armenian	96290
Probable	First/main language not English	13w5.00	Main spoken language Quechua	96317
Probable	First/main language not English	13uv.00	Main spoken language Maltese	96370
Probable	First/main language not English	13uu.00	Main spoken language Malay	96376
Probable	First/main language not English	13le.00	Main spoken language Brawa	96485
Probable	First/main language not English	13wM.00	Main spoken language Tibetan	96558
Probable	First/main language not English	13uG.00	Main spoken language Burmese	96559
Probable	First/main language not English	13uT.00	Main spoken language Finnish	96560
Probable	First/main language not English	13us.00	Main spoken language Macedonian	96611
Probable	First/main language not English	13wN.00	Main spoken language Tongan	96634
Probable	First/main language not English	13wT.00	Main spoken language Uzbek	96784
Probable	First/main language not English	13u2.00	Main spoken language Oromo	96857
Probable	First/main language not English	13u4.00	Main spoken language Afar	96868
Probable	First/main language not English	13wa.00	Main spoken language Zulu	96873
Probable	First/main language not English	13uQ.00	Main spoken language Estonian	96928
Probable	First/main language not English	13uk.00	Main spoken language Kashmiri	97015
Probable	First/main language not English	13uz.00	Main spoken language Mongolian	97038
Probable	First/main language not English	13wW.00	Main spoken language Wolof	97039
Probable	First/main language not English	13wQ.00	Main spoken language Turkmen	97041
Probable	First/main language not English	13u9.00	Main spoken language Azerbaijani	97083
Probable	First/main language not English	13wH.00	Main spoken language Sundanese	97131
Probable	First/main language not English	13uX.00	Main spoken language Georgian	97212
Probable	First/main language not English	13wX.00	Main spoken language Xhosa	97273

Probable	First/main language not English	13uc.00	Main spoken language Indonesian	97274
Probable	First/main language not English	13w3.00	Main spoken language Oriya	97297
Probable	First/main language not English	9NUC.11	Persian language interpreter needed	97298
Probable	First/main language not English	13wB.00	Main spoken language Southern Sotho	97439
Probable	First/main language not English	13w6.00	Main spoken language Romansh	97440
Probable	First/main language not English	13uB.00	Main spoken language Basque	97574
Probable	First/main language not English	13ul.00	Main spoken language Kazakh	97595
Probable	First/main language not English	9NUz.00	Bulgarian language interpreter needed	97644
Probable	First/main language not English	13wP.00	Main spoken language Tsonga	97685
Probable	First/main language not English	13uK.00	Main spoken language Catalan	97997
Probable	First/main language not English	9Nmm.00	Burmese language interpreter needed	98062
Probable	First/main language not English	13uZ.00	Main spoken language Guarani	98070
Probable	First/main language not English	13up.00	Main spoken language Lao	98132
Probable	First/main language not English	13um.00	Main spoken language Kinyarwanda	98194
Probable	First/main language not English	13uy.00	Main spoken language Moldavian	98215
Probable	First/main language not English	13uw.00	Main spoken language Maori	98255
Probable	First/main language not English	13w4.00	Main spoken language Filipino	98285
Probable	First/main language not English	13uY.00	Main spoken language Kalaallisut	98510
Probable	First/main language not English	13ub.00	Main spoken language Icelandic	98604
Probable	First/main language not English	13u8.00	Main spoken language Aymara	98762
Probable	First/main language not English	9NmQ.00	Hungarian language interpreter needed	98809
Probable	First/main language not English	9NUy.00	Romanian language interpreter needed	98841
Probable	First/main language not English	13wS.00	Main spoken language Uighur	99712
Probable	First/main language not English	9Nn1.00	Tsonga language interpreter needed	99794
Probable	First/main language not English	13ui.00	Main spoken language Javanese	100011
Probable	First/main language not English	13uL.00	Main spoken language Slovak	100013
Probable	First/main language not English	9NmA.00	Macedonian language interpreter needed	100438
Probable	First/main language not English	13uR.00	Main spoken language Faeroese	100707
Probable	First/main language not English	13uS.00	Main spoken language Fijian	100714
Probable	First/main language not English	13ug.00	Main spoken language Inuktitut	100716
Probable	First/main language not English	13uW.00	Main spoken language Galician	100743
Probable	First/main language not English	9Nn7.00	Slovenian language interpreter needed	100759
Probable	First/main language not English	13uH.00	Main spoken language Belarusian	100828
Probable	First/main language not English	13uD.00	Main spoken language Bihari	100949
Probable	First/main language not English	13wb.00	Main spoken language Konkani	101038
Probable	First/main language not English	13uJ.00	Main spoken language Central Khmer	101189
Probable	First/main language not English	13wA.00	Main spoken language Dari	101220
Probable	First/main language not English	9Nn4.00	Telugu language interpreter needed	101614
Probable	First/main language not English	13ut.00	Main spoken language Malagasy	101620
Probable	First/main language not English	13ud.00	Main spoken language Interlingua	101659
Probable	First/main language not English	13l9.11	Main spoken language Iban	101761
Probable	First/main language not English	13uM.00	Main spoken language Corsican	101788
Probable	First/main language not English	9NUc.11	Punjabi language interpreter needed	101814
Probable	First/main language not English	13uY.11	Main spoken language Greenlandic	102007
Probable	First/main language not English	13uC.00	Main spoken language Dzongkha	102127
Probable	First/main language not English	13uF.00	Main spoken language Breton	102128
Probable	First/main language not English	13ue.00	Main spoken language Interlingue	102129
Probable	First/main language not English	13u3.00	Main spoken language Abkhazian	102184
Probable	First/main language not English	13u7.00	Main spoken language Assamese	102218
Probable	First/main language not English	13uA.00	Main spoken language Bashkir	102259
Probable	First/main language not English	13wC.00	Main spoken language Tswana	102877
Probable	First/main language not English	13uV.00	Main spoken language Frisian	103200
Probable	First/main language not English	13w7.00	Main spoken language Samoan	103219
Probable	First/main language not English	13uq.00	Main spoken language Bamun	104071
Probable	First/main language not English	9NmC.00	Latvian language interpreter needed	104635
Probable	First/main language not English	13wE.00	Main spoken language Ndebele	104886
Probable	First/main language not English	9Nm6.00	Brawa language interpreter needed	104901
Probable	First/main language not English	9Nmd.00	Catalan language interpreter needed	105079
Probable	First/main language not English	13wV.00	Main spoken language Tetum	105523
Probable	First/main language not English	13wc.00	Main spoken language Aragonese	105529
Probable	First/main language not English	13wJ.00	Main spoken language Tajik	105960
Probable	First/main language not English	13Z6900	First language not English	108098
Probable	First/main language not English	9Nn6.00	Turkmen language interpreter needed	108184
Probable	First/main language not English	9NmE.00	Kinyarwanda language interpreter needed	109489
Probable	First/main language not English	13l6.11	Main spoken language Gujarati	109896
Probable	First/main language not English	13wF.00	Main spoken language Swati	111709
Probable	First/main language not English	13w0.00	Main spoken language Nauru	111734
Probable	First/main language not English	9Nm1.00	Moldavian language interpreter needed	111788
Probable	First/main language not English	13wZ.00	Main spoken language Zhuang	111789
Probable	First/main language not English	13wY.00	Main spoken language Yiddish	111874
Probable	First/main language not English	13w8.00	Main spoken language Sango	111919
Probable	First/main language not English	9NnC.00	Sundanese language interpreter needed	112222
Probable	First/main language not English	13uo.00	Main spoken language Rundi	112260

Probable	First/main language not English	13wK.00	Main spoken language Tatar	112321
Probable	First/main language not English	13lD.00	Main spoken language Portuguese	32728
Possible	Non-UK origin	1343	Asian origin	25801
Possible	Non-UK origin	1345	South American origin	32101
Possible	Non-UK origin	2263.11	O/E - Asian origin	32132
Possible	Non-UK origin	1344	North American origin	41150
Possible	Non-UK origin	1342	African origin	45125
Possible	Non-UK origin	134A.00	West Indian origin	45131
Possible	Non-UK origin	1347	Indian origin	45144
Possible	Non-UK origin	1348	Middle Eastern origin	47951
Possible	Non-UK origin	9SA5.00	Other African countries (NMO)	47969
Possible	Non-UK origin	1346	Australian origin	47975
Possible	Non-UK origin	2263	O/E - Mongoloid origin	66536

Table S3. Number and percentage of recorded migrants in CPRD GOLD per year by certainty of migration status (1997-2018).

Year	Migrants		Definite migrants		Probable migrants		Possible migrants		Definite + Probable migrants	
	n/N	%	n/N	%	n/N	%	n/N	%	n/N	%
**Total**	403,768/16,071,111	2·51	178,749/16,071,111	1·11	216,731/16,071,111	1·35	8,288/16,071,111	0·05	395,480/16,071,111	2·46
**1997**	4,417/2,210,551	0·20	772/2,210,551	0·0349	3,392/2,210,551	0·153	253/2,210,551	0·0114	4,164/2,210,551	0·00188
**1998**	6924/2,662,706	0·26	1,949/2,662,706	0·0732	4,604/2,662,706	0·173	371/2,662,706	0·0139	6,553/2,662,706	0·246
**1999**	9329/3,281,113	0·28	2,955/3,281,113	0·0901	5,934/3,281,113	0·181	440/3,281,113	0·0134	8,889/3,281,113	0·271
**2000**	12,291/3,787,586	0·33	4,197/3,787,586	0·111	7,555/3,787,586	0·199	539/3,787,586	0·0142	11,752/3,787,586	0·310
**2001**	17,176/4,362,237	0·39	6,393/4,362,237	0·158	9,461/4,362,237	0·217	822/4,362,237	0·0188	16,354/4,362,237	0·375
**2002**	23,638/4,812,744	0·49	10,911/4,812,744	0·227	11,742/4,812,744	0·244	985/4,812,744	0·0205	22,653/4,812,744	0·471
**2003**	31,637/5,194,514	0·609	16,372/5,194,514	0·315	14,132/5,194,514	0·272	1,133/5,194,514	0·0218	30,504/5,194,514	0·587
**2004**	42,869/5,435,276	0·79	23,824/5,435,276	0·438	17,679/5,435,276	0·325	1,366/5,435,276	0·0251	41,503/5,435,276	0·764
**2005**	53,229/5,534,090	0·96	29,208/5,534,090	0·528	22,357/5,534,090	0·404	1,664/5,534,090	0·0301	51,565/5,534,090	0·932
**2006**	63,387/5,653,376	1·12	22,431/5,653,376	0·591	27,654/5,653,376	0·489	2,302/5,653,376	0·0407	61,085/5,653,376	1·08
**2007**	71,423/5,716,075	1·25	34,474/5,716,075	0·603	34,50/5,716,075	0·604	2,440/5,716,075	0·0427	68,983/5,716,075	1·21
**2008**	83,092/5,682,548	1·46	38,150/5,682,548	0·671	42,415/5,682,548	0·746	2,527/5,682,548	0·0445	80,565/5,682,548	1·42
**2009**	102,292/5,700,405	1·79	41,733/5,700,405	0·732	58,246/5,700,405	1·02	2,313/5,700,405	0·0406	99,979/5,700,405	1·75
**2010**	121,683/5,694,007	2·14	45,192/5,694,007	0·794	74,034/5,694,007	1·30	2,457/5,694,007	0·0432	119,226/5,694,007	2·09
**2011**	137,231/5,583,145	2·46	46,943/5,583,145	0·841	87,862/5,583,145	1·57	2,426/5,583,145	0·0435	134,805/5,583,145	2·41
**2012**	155,439/5,490,938	2·83	50,303/5,490,938	0·916	102,603/5,490,938	1·87	2,533/5,490,938	0·0461	152,906/5,490,938	2·78
**2013**	165,392/5,427,745	3·05	56,225/5,427,745	1·04	106,300/5,427,745	1·96	2,867/5,427,745	0·0528	162,525/5,427,745	2·99
**2014**	151,699/5,094,287	2·98	58,425/5,094,287	1·15	90,520/5,094,287	1·78	2,754/5,094,287	0·0541	148,945/5,094,287	2·92
**2015**	139,348/4,484,174	3·11	55,934/4,484,174	1·25	81,960/4,484,174	1·83	1,454/4,484,174	0·0324	137,894/4,484,174	3·08
**2016**	106,614/3,600,079	2·96	39,106/3,600,079	1·09	66,553/3,600,079	1·85	955/3,600,079	0·0265	105,659/3,600,079	2·93
**2017**	94,293/3,075,162	3·07	29,888/3,075,162	0·972	63,474/3,075,162	2·06	931/3,075,162	0·0303	93,362/3,075,162	3·04
**2018**	100,626/2,761,397	3·64	38,096/2,761,397	1·38	61,688/2,761,397	2·23	842/2,761,397	0·0305	99,784/2,761,397	3·61

Table S4. Demographic characteristics of recorded migrants in CPRD GOLD at the time of the 2011 census by certainty of migration status (England and Wales).

Demographic characteristic		Migrants (%)	Definite migrants (%)	Probable migrants (%)	Possible migrants (%)	Definite + Probable migrants (%)
**Total**		107,526 (100%)	36,471 (33·9%)*	68,983 (64·2%)	2,072 (1·92%)	105,454 (98·1%)
**Sex**	Male	51,252 (47·7%)	17,894 (49·1%)	32,325 (46·9%)	1,033 (49·9%)	50,219 (47·6%)
	Female	56,274 (52·3%)	18,577 (50·9%)	36,658 (53·1%)	1,039 (50·1%)	55,235 (52·4%)
**Year of birth**	1900-1919	119 (0·11%)	36 (0·10%)	77 (0·112%)	6 (0·290%)	113 (0·107%)
	1920-1939	4,560 (4·24%)	1,230 (3·37%)	3,235 (4·69%)	95 (4·58%)	4,465 (4·23%)
	1940-1959	13,267 (12·3%)	4,332 (11·9%)	8,616 (12·5%)	319 (15·4%)	12,948 (12·3%)
	1960-1979	39,600 (36·8%)	15,977 (43·8%)	22,722 (32·9%)	901 (43·5%)	38,699 (36·7%)
	1980-1999	35,886 (33·4%)	12,414 (34·0%)	22,875 (33·2%)	597 (28·8%)	35,289 (33·5%)
	2000-2018	14,094 (13·1%)	2,482 (6·81%)	11,458 (16·6%)	154 (7·43%)	13,940 (13·2%)
**Ethnicity**	White British	1,667 (1·55%)	840 (2·30%)	796 (1·15%)	31 (1·50%)	1,636 (1·55%)
	White Non-British	34,821 (32·4%)	11,568 (31·7%)	23,168 (33·6%)	85 (4·10%)	34,736 (32·9%)
	Mixed	2,494 (2·32%)	1,057 (2·90%)	1,367 (1·98%)	70 (3·38%)	2,424 (2·30%)
	Asian/Asian British	39,845 (37·1%)	8,419 (23·1%)	30,606 (44·4%)	820 (39·6%)	39,025 (37·0%)
	Black/African/Caribbean/Black British	10,859 (10·1%)	5,782 (15·9%)	4,563 (6·64%)	514 (24·8%)	10,345 (9·81%)
	Other	8,632 (8·03%)	3,130 (8·58%)	5,371 (7·82%)	131 (6·32%)	8,501 (8·06%)
	Unknown	9,208 (8·56%)	5,675 (15·6%)	3,112 (4·51%)	421 (20·3%)	8,787 (8·33%)
**Geographical region of birth (WHO Region)**	African Region	5,617 (5·22%)	5,617 (15·4%)	··	··	··
	European Region	10,126 (9·42%)	10,126 (27·8%)	··	··	··
	Eastern Mediterranean Region	2,970 (2·76%)	2,970 (8·14%)	··	··	··
	Region of the Americas	2,229 (2·07%)	2,229 (6·11%)	··	··	··
	South East Asian Region	4,088 (3·80%)	4,088 (11·2%)	··	··	··
	Western Pacific Region	3,622 (3·37%)	3,622 (9·93%)	··	··	··
	Unknown	78,742 (73·2%)	7,819 (21·4%)	··	··	··
**Geographical region of birth (continent)**	Africa	5,617 (5·22%)	5,617 (15·4%)	··	··	··
	Europe	10,048 (9·34%)	10,048 (27·6%)	··	··	··
	Middle East & Asia	10,022 (9·32%)	10,022 (27·5%)	··	··	··
	The Americas & Caribbean	2,229 (2·07%)	2,229 (6·11%)	··	··	··
	Antarctica & Oceania	845 (0·79%)	845 (2·3%)	··	··	··
	Unknown	78,737 (73·2%)	7,710 (21·1%)	··	··	··

**Practice region**	London	46,043 (42·8%)	21,324 (58·5%)	25,301 (34·1%)	1,218 (58·8%)	44,825 (42·5%)
	South Central	10,641(9·90%)	3,866 (10·6%)	6,566 (9·56%)	209 (10·1%)	10,432 (9·89%)
	South East Coast	6,615 (6·15%)	1,483 (4·06%)	5,042 (7·31%)	90 (4·34%)	6,525 (6·19%)
	North West	11,538 (10·7%)	3,197 (8·77%)	8,258 (12·0%)	83 (4·01%)	11,455 (10·9%)
	West Midlands	10,647 (9·93%)	1,190 (3·26%)	9,347 (13·5%)	110 (5·31%)	10,537 (10·0%)
	East of England	8,584 (7·98%)	1,445 (3·96%)	7,069 (10·2%)	70 (3·38%)	8,514 (8·07%)
	South West	6,039 (5·62%)	2,299 (6·30%)	3,709 (5·40%)	31 (1·50%)	6,008 (5·70%)
	North East	2,370 (2·20%)	154 (0·422%)	2,209 (3·20%)	7 (0·338%)	2,363 (2·24%)
	East Midlands	1,770 (1·65%)	150 (0·422%)	1,541 (2·24%)	79 (3·81%)	1,691 (1·60%)
	Yorkshire & The Humber	760 (0·707%)	101 (0·277%)	574 (0·83%)	85 (4·10%)	675 (0·64%)
	Wales	2,519 (2·34%)	1,262 (3·46%)	1,167 (1·70%)	90 (4·34%)	2,429 (2·30%)

*Percentages are calculated across columns except for the first row which are percentages.

.

Table S5. Migrants as a percentage of all patients registered in a CPRD GOLD practice region (2009-2018).

Practice region	No. recorded migrants	No. registered patients	Percentage migrants in region
North East	4,980	205,916	2·42
North West	31,964	1,534,366	2·08
Yorkshire & The Humber	1,740	483,845	0·36
East Midlands	4,594	492,978	0·93
West Midlands	29,629	1,280,599	2·31
East of England	24,006	1,319,786	1·82
South West	19,734	1,322,423	1·49
South Central	48,740	1,674,896	2·91
London	171,368	2,302,173	7·44
South East Coast	43,089	1,625,941	2·65
Northern Ireland	921	434,498	0·21
Scotland	12,135	1,762,543	0·69
Wales	10,868	1,631,147	0·67

Table S6. Percentage of individuals recorded as migrants in CPRD GOLD and ONS country of birth (2004-2018).

Year	Percentage of CPRD GOLD patients recorded as international migrants (95% CI)	Percentage of individuals living in the UK recorded by ONS as international migrants	Ratio of total percentages (% CPRD / % ONS)
2004	0·79 (0·781 - 0·796)	8·89	0·09
2005	0·96 (0·954 - 0·970)	9·36	0·10
2006	1·12 (1·113 - 1·130)	10·05	0·11
2007	1·25 (1·240 - 1·259)	10·59	0·12
2008	1·46 (1·452 - 1·472)	11·09	0·13
2009	1·79 (1·784 - 1·805)	11·42	0·16
2010	2·14 (2·125 - 2·149)	11·68	0·18
2011	2·46 (2·445 - 2·471)	12·26	0·20
2012	2·83 (2·817 - 2·845)	12·44	0·23
2013	3·05 (3·033 - 3·062)	12·52	0·24
2014	2·98 (2·963 - 2·993)	13·00	0·23
2015	3·11 (3·092 - 3·124)	13·33	0·23
2016	2·96 (2·944 - 2·979)	14·14	0·21
2017	3·07 (3·047 - 3·086)	14·39	0·21
2018	3·64 (3·622 - 3·666)	14·24	0·26

Table S7. Percentage of CPRD patients recorded as migrants and ONS 2011 census percentage of migrants in the population by age band (2011).

Age band in years	CPRD GOLD migrants / all individuals (%)	ONS 2011 census migrants / all individuals (%)	Ratio of total percentages (% CPRD / % ONS)
**0-15**	17,838 / 745,500 (2·4%)	594,415 / 10,579,132 (5·6%)	0·43
**16-24**	11,129 / 446,584 (2·5%)	896,719 / 6,658,636 (13·5%)	0·18
**25-34**	30,178 / 552,426 (5·5%)	1,892,699 / 7,520,524 (25·2%)	0·22
**35-49**	28,135 / 941,841 (3·0%)	2,004,537 / 11,931,776 (16·8%)	0·18
**50-64**	12,586 / 810,017 (1·6%)	1,239,056 /10,162,771 (12·2%)	0·13
**65-74**	4,194 / 397,473 (1·1%)	479,211 / 4,852,833 (9·9%)	0·11
**75-84**	2,684 / 256,482 (1·1%)	300,122 / 3,115,552 (9·6%)	0·11
**85 plus**	782 /118,357 (0·7%)	98,251 / 1,254,688 (7·8%)	0·09

Table S8. Age breakdown of migrants in CPRD GOLD and in ONS at the time of the 2011 census.

Age band in years	CPRD GOLD		ONS 2011 census	
	No. migrants	% of migrants	No. migrants	% of migrants
**All age bands**	107,526	100	7,505,010	100
**0-15**	17,838	16·6	594,415	7·92
**16-24**	11,129	10·4	896,719	12·0
**25-34**	30,178	28·07	1,892,699	25·2
**35-49**	28,135	26·2	2,004,537	26·7
**50-64**	12,586	11·7	1,239,056	16·5
**65-74**	4,194	3·90	479,211	6·39
**75-84**	2,684	2·50	300,122	4·00
**85 plus**	782	0·73	98,251	1·31

Table S9. Breakdown of migrants recorded in CPRD GOLD at the time of the ONS 2011 census by the 18 group classification of ethnicity (2011).

Ethnic group	Number of migrants	% of migrants
All migrants	107,526	100
White British	1,667	1·55
White Irish	551	0·51
White Other	28,605	26·6
White NOS	5,665	5·27
Mixed White and Black	765	0·71
Mixed White and Asian	474	0·44
Mixed Asian and Black	141	0·13
Mixed Other	1,056	0·98
Mixed NOS	58	0·05
Indian	13,283	12·4
Pakistani	10,084	9·38
Bangladeshi	3,935	3·66
Other Asian	10,028	9·33
Black Caribbean	1,158	1·08
Black African	8,582	7·98
Black Other	1,119	1·04
Chinese	2,515	2·34
Other ethnic group	8,632	8·03
Unknown	9,208	8·56
